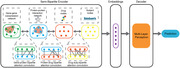# PreSiBOGNN: Multi‐Modal Graph Neural Network for Prediction of Cognitive Improvement in Medication Usage in UK Biobank

**DOI:** 10.1002/alz.089290

**Published:** 2025-01-09

**Authors:** Dhawal Priyadarshi, Heng Huang, Nathan J Sahelijo, Reza Shirkavand, Gyungah R Jun

**Affiliations:** ^1^ Boston University Chobanian & Avedisian School of Medicine, Boston, MA USA; ^2^ Department of Computer Science at the University of Maryland College Park, College Park, MD USA; ^3^ Department of Biostatistics, Boston University School of Public Health, Boston, MA USA; ^4^ Department of Ophthalmology, Boston University Chobanian & Avedisian School of Medicine, Boston, MA USA; ^5^ Department of Medicine (Biomedical Genetics), Boston University Chobanian & Avedisian School of Medicine, Boston, MA USA

## Abstract

**Background:**

The prohibitive costs of drug development for Alzheimer’s Disease (AD) emphasize the need for alternative in silico drug repositioning strategies. Graph learning algorithms, capable of learning intrinsic features from complex network structures, can leverage existing databases of biological interactions to improve predictions in drug efficacy. We developed a novel machine learning framework, the PreSiBOGNN, that integrates muti‐modal information to predict cognitive improvement at the subject level for precision medicine in AD.

**Method:**

The graph neural network framework integrates four layers of input data including transcriptome, proteome, drug, and subject to connect to bipartite graphs in the United Kingdom Biobank (UKBB). Medication usage, clinical, and GWAS data were downloaded for 48187 subjects with first and second cognitive exams from the UKBB. The protein and transcriptome layers were constructed using the String database and gene coexpression networks generated from single nuclei RNA data (Sahelijo et al. 2022). Layers were connected by binary bipartite graphs constructed using drug information from the UniProt and DrugCentral databases. Sequential Graph Attention Networks convoluted embedded features generated by each layer in a hierarchical order: 1. gene‐gene, 2. gene‐protein, 3. protein‐protein, 4. protein‐drug, 5. drug‐subj. Feature embeddings were decoded using a multilayer perceptron to predict cognitive improvement between the first and second cognitive exams. Two models compared methods of data aggregation. The first model follows a strict hierarchy, aggregating inter‐layer data in a single direction (gene > protein > drug > subj). The second model allows for fluid message passing between inter‐layers. We used 60% of the UKBB subjects for training and 30% for validation. We assessed the model with the greatest training accuracy using the remaining 10% of subjects.

**Result:**

We observed that the performance of the strict message‐passing model attained validation and test accuracy of 57.7% and 51.2%, respectively. Performance of the fluid message passing model improved prediction accuracy with 61.3% and 58.1% in the test set.

**Conclusion:**

Our investigation suggests the feasibility of the PreSiBOGNN framework to infer cognitive improvement of existing drugs by integrating medication, multi‐omics, and clinical data. Future work will focus on model optimizations and the integration of additional modalities including compound‐specific fingerprint data.